# Childhood Adversity and Adolescent Smartphone Use Across Sexual Orientation and Gender Expression

**DOI:** 10.1001/jamanetworkopen.2024.6448

**Published:** 2024-04-12

**Authors:** Xinyu Zheng, Weiqing Jiang, Shuyi Peng, Qianyu Liu, Yitong He, Cuihong Huang, Yilin Hua, Ciyong Lu, Lan Guo

**Affiliations:** 1 Department of Medical Statistics and Epidemiology, School of Public Health, Sun Yat-sen University, Guangzhou, People’s Republic of China

## Abstract

**Importance:**

Nonheterosexual and gender-nonconforming (GNC) individuals tend to report adverse childhood experiences (ACEs) more frequently compared with heterosexual and gender-conforming individuals, and individuals who have experienced ACEs, identify as nonheterosexual, or exhibit moderate to high levels of GNC are more prone to engaging in problematic smartphone use (PSU). However, there is limited school-based data among adolescents regarding this matter.

**Objectives:**

To explore the associations between ACEs and PSU among adolescents across different sexual orientation and gender expression groups.

**Design, setting, and participants:**

Using data from the 2021 School-Based Chinese Adolescents Health Survey, this cross-sectional study includes participants from 288 public high schools across 8 provinces in China. Statistical analysis was performed from October 2023 to February 2024.

**Exposures:**

Data on ACEs, sexual orientations, and gender expressions (high, moderate, and low GNC) were collected.

**Main outcomes and measures:**

PSU was assessed using the 10-item Smartphone Addiction Scale-Short Version (SAS-SV). Weighted linear, logistic, or Poisson regression models were used.

**Results:**

Among the 85 064 adolescents included (mean [SD] age, 14.92 [1.77] years), 42 632 (50.1%) were female, 70 157 (83.2%) identified as Han Chinese, and 14 208 (16.8) identified as other ethnicities (Miao, Hui, Yi, Dai, and other ethnic groups). The prevalence of PSU among participants was 35.4%. Weighted Poisson regression models indicated that the interaction between GNC and ACE was significant (adjusted prevalence ratio [APR], 0.98; 95% CI, 0.97-0.99). Further stratified analysis demonstrated homosexual adolescents who experienced 4 or more ACEs showed a significantly increased prevalence of PSU (APR, 1.79; 95% CI, 1.64-1.96). Similarly, a markedly higher prevalence of PSU was observed among bisexual individuals with 4 or more ACEs (APR, 1.60; 95% CI, 1.41-1.80). Regarding gender expression categories, a significantly higher prevalence of PSU was noted among high GNC adolescents with 4 or more ACEs (APR, 1.78; 95% CI, 1.60-1.98) compared with low GNC adolescents without ACEs. Furthermore, experiencing any 3 ACE categories (abuse, neglect, and household dysfunction) was associated with an increased prevalence of PSU across different sexual orientation and gender expression subgroups.

**Conclusions and relevance:**

In this cross-sectional study, the amalgamation of elevated ACE scores with nonheterosexual orientations or GNC identities was significantly associated with increased PSU prevalence. These findings underscore that preventing ACEs may be beneficial in mitigating PSU among adolescents, particularly for nonheterosexual adolescents and those with high levels of GNC.

## Key Points

**Question:**

Do the associations between adverse childhood experiences (ACEs) and problematic smartphone use (PSU) among adolescents vary across sexual orientation and gender expression groups?

**Findings:**

In this cross-sectional study involving 85 064 adolescents in China, sexual minorities and gender-nonconforming (GNC) individuals were more likely to have experienced ACEs; those exposed to ACEs demonstrated an increased susceptibility to engaging in PSU, irrespective of their sexual orientations or gender expressions. Moreover, a significantly higher prevalence of PSU was noted when higher ACE scores were combined with either a nonheterosexual orientation or a GNC identity.

**Meaning:**

These findings support the potential benefits of implementing preventive interventions aimed at addressing ACEs among adolescents, especially for those identifying as nonheterosexual or GNC.

## Teaser

This cross-sectional study evaluates the associations between adverse childhood experiences and problematic smartphone use among adolescents in China across different sexual orientation and gender expression groups.

## Introduction

The widespread adoption of smartphones among adolescents globally has brought about a surge in their usage, yet alongside this trend emerges a concerning phenomenon known as problematic smartphone use (PSU).^1^ PSU is characterized by excessive and inappropriate smartphone usage, often coupled with an inability to regulate usage, leading to adverse outcomes among adolescents, such as mental health issues,^2^ academic struggles,^3^ and interpersonal challenges.^4^ According to the compensatory internet use theory, individuals may resort to excessive smartphone use as a coping mechanism to escape stressful situations and fulfill unmet needs that cannot be addressed in the physical realm.^5,6^

Adverse childhood experiences (ACEs), also known as childhood adversity, encompass a broad range of potentially stressful events during childhood, including abuse, neglect, and household dysfunction.^7^ These stressful experiences are increasingly acknowledged as risk factors for various social and health-related problems,^8,9^ including PSU among adolescents.^10^ However, existing research often focuses on the general population, fails to distinguish between heterosexual and nonheterosexual individuals, and overlooks gender expressions.^11,12^ Recent research has highlighted a higher prevalence of PSU among individuals identifying as nonheterosexual or exhibiting gender nonconformity (GNC, indicating a misalignment between one’s gender expression and societal expectations of femininity or masculinity) compared with heterosexual individuals or those conforming to societal gender norms.^13,14^ Drawing from the minority stress model, nonheterosexual and GNC individuals often encounter various minority stressors (eg, prejudice, discrimination, harassment, and violence) in their daily lives.^15,16^ Consequently, PSU among sexual minority and GNC youths may manifest as a maladaptive coping mechanism in response to experiences of social and minority stress. Similarly, ACEs could also be viewed as traumatic stressors that render these populations more susceptible to PSU.^10^ Recent research indicates that nonheterosexual individuals report ACEs more frequently, particularly instances of abuse and household dysfunction.^17^ Moreover, GNC individuals are significantly more likely to report a surplus of ACEs than individuals who conform to societal gender expectations.^18,19^

Despite evidence indicating that nonheterosexual and GNC individuals are more prone to experiencing childhood adversities or PSU challenges independently, limited research has explored the intricate associations between ACEs and PSU across sexual orientation and gender expression groups. Furthermore, there is a lack of nationally representative samples of school-based Chinese adolescents encompassing adequate subgroups of sexual orientations and gender expressions to examine these associations comprehensively. Therefore, this study aims to address this gap by investigating the associations between ACEs and PSU across different subgroups of sexual orientation and gender expression among Chinese adolescents.

## Methods

### Study Design and Population

The study used data from the 2021 School-Based Chinese Adolescents Health Survey (SCAHS), an ongoing survey examining health-related behaviors among Chinese adolescents in grades 7 through 12.^20^ SCAHS has conducted large-scale cross-sectional data collection every 2 years since 2007, along with longitudinal data collection between 2009 and 2012. Self-reported questionnaires were administered in classrooms.^21^ The 2021 SCAHS used a multistage, stratified cluster, random sampling approach. Detailed data collection procedures are outlined in the eMethods in Supplement 1. A total of 85 611 students from 1728 classes across 288 public high schools were invited to participate. Of these, 85 046 completed and submitted the questionnaire, resulting in a response rate of 99.3%. Participation in the SCAHS study was voluntary, with all participants and 1 of their legal guardians providing informed consent. Ethical approval for the study was obtained from the Sun Yat-sen University School of Public Health institutional review board. This study adhered to the Strengthening the Reporting of Observational Studies in Epidemiology (STROBE) reporting guidelines.

### ACEs

To capture ACEs, we extracted 14 forms of ACEs from the self-reported questionnaires, including abuse (sexual abuse, physical abuse, and emotional abuse), neglect (physical neglect and emotional neglect), and household dysfunction (parental separation or divorce, household criminality, household domestic violence, household mental illness, household substance abuse, family financial problems, death of parent, witness of community violence, and sex discrimination). The detailed questionnaire items and definitions of each ACE indicator are available in the eMethods in Supplement 1. Responses to each item were dichotomized and summed to generate a cumulative ACE score for each participant, ranging from 0 to 14. We further categorized participants into 5 groups based on the cumulative ACE scores: 0, 1, 2, 3, and 4 or higher.^22^

### Sexual Orientation and Gender Expression

Sexual orientation was assessed using the question “Which gender do you think you are romantically attracted to?” with response options including male, female, both male and female, not sure, unwilling to answer, and neither men nor women.^23^ Students were categorized as heterosexual, homosexual, bisexual, and not sure based on their response and biological sex. Heterosexual was defined as male students who describe themselves as romantically attracted to female students and female students who describe themselves as romantically attracted to male students. Homosexual was defined as male students who describe themselves as romantically attracted to male students and female students who describe themselves as romantically attracted to female students. Bisexual was defined as students who describe themselves as romantically attracted to both male and female students. In this analysis, the variable of sexual orientation was dichotomized into heterosexual adolescents and nonheterosexual adolescents, and the reference group was heterosexual adolescents.

Gender expression was measured with a validated measure by asking the following question: “A person’s appearance, style, dress, or the way they walk or talk may affect how people describe them. How do you think people at school would describe you?”^24^. We adopted a 3-level GNC variable based on previous studies, including one of our previous works^19,25^: (1) high GNC (somewhat, mostly, and very masculine female students and somewhat, mostly, and very feminine male students), (2) moderate GNC (equally feminine and masculine female and male students), and (3) low GNC (somewhat, mostly, and very feminine female students and somewhat, mostly, and very masculine male students). Low GNC was defined as female students who describe themselves as very/mostly/somewhat feminine and male students who describe themselves as very/mostly/somewhat masculine. Moderate GNC was defined as students who describe themselves as equally feminine and masculine. High GNC was defined as female students who describe themselves as very/mostly/somewhat masculine and male students who describe themselves as very/mostly/somewhat feminine. In this analysis, the variable of gender nonconformity was categorized into 2 groups: moderate or high GNC adolescents and low GNC adolescents. The reference group was low GNC adolescents.More details can be found in the eMethods in Supplement 1.

### PSU

PSU was measured using the 10-item smartphone addiction scale short version (SAS-SV),^26^ which assesses 4 of the 6 components of a widely used 6-component model of addiction: conflict, withdrawal, tolerance, and salience.^27^ Respondents selected options on a 6-point Likert scale ranging from 6, which indicated strongly disagree, to 1, which indicated strongly agree, with statements such as “Missing planned work due to smartphone use.” The sum of the items provides an overall SAS-SV score (range: 10-60), with a higher score indicating more severe PSU. Male students with SAS-SV scores 31 and higher and female students with SAS-SV scores 33 and higher were classified as the PSU group.^28^ The SAS-SV exhibited high internal consistency in the present study, with a Cronbach α of 0.88.

### Covariates

To identify potential confounding factors, we used directed acyclic graphs to delineate the most well-established associations among the variables under consideration in our study (eFigure in Supplement 1). Age, ethnicity (categorized as either Han or others, with participants of other ethnic groups requested to indicate their respective ethnicity; this list included Miao, Hui, Yi, Dai, and other ethnic groups), and place of residence were collected through self-reported questionnaires. In this nationally conducted school-based study in China, we gathered information on ethnicity as a demographic characteristic.

### Statistical Analysis

Data were presented as mean (SD) for continuous variables and as frequencies with percentages for categorical variables. Comparison of characteristics across different sexual orientation and gender expression groups used analysis of variance for continuous variables and χ^2^ tests for categorical variables. Prevalence estimates of PSU and corresponding 95% CIs were calculated based on sample characteristics, using Taylor series linearization to account for the complex sample design, including stratification, clustering, and weighting.^29^ Initially, we performed weighted linear and logistic regression models that accounted for the stratified cluster survey design and nonresponses to assess the associations between sexual orientation and gender expression and ACEs, using heterosexual or low GNC adolescents as the reference group, respectively. Linear regression models were applied for the continuous outcome (the total number of ACEs), while logistic regressions were used for the binary outcome (presence or absence of each ACE category). Moreover, to investigate associations between ACEs and PSU, prevalence ratios (PRs) with 95% CIs were estimated using a weighted Poisson regression model that adjusted for the stratified cluster survey design instead of logistic regression models due to the relatively common occurrence of PSU.^30,31^ Additionally, to assess multiplicative interactions between ACEs and sexual orientation and gender expression on PSU, we introduced a product interaction term (ACEs × sexual orientation / ACEs × gender expression) into the adjusted Poisson regression models. The PR (95% CI) and *P*-value of the product term were used to measure interaction on the multiplicative scale. Subsequently, stratified analyses were conducted to assess whether the association between ACEs and PSU varied by sexual orientation and gender expression. Specifically, PRs were calculated for the association of the number of ACEs (0, 1, 2, 3, ≥4) and each of the 3 ACE categories (abuse, neglect, and household dysfunction) with PSU across sexual orientation and gender expression categories. Heterosexual students with an ACE score of 0, heterosexual students not reporting in the ACE category in question, low GNC adolescents with an ACE score of 0, and low GNC adolescents not reporting in the ACE category in question served as reference groups, respectively. Statistical analyses were conducted using Stata, version 17.0 (StataCorp LLC). All *P* values were 2-sided, and statistical significance was set at *P* < .05. Analyses were performed from October 2023 to February 2024.

## Results

### Participant Characteristics

Among the 85 046 participants included, 42 632 (50.1%) were female, 70 157 (83.2%) identified as Han Chinese, 14 208 (16.8%) identified as other ethnicities (Miao, Hui, Yi, Dai, and other ethnic groups), and the mean (SD) age was 14.92 (1.77) years (Table 1). The characteristics of the study population, stratified by sexual orientation groups, are depicted in Table 1, while eTable 1 in Supplement 1 presents the characteristics stratified by gender expressions. Excluded participants with missing data, although exhibiting relatively minor differences, tended to be younger, more frequently female, from other ethnic backgrounds, residing in rural areas, and reported higher levels of neglect and household dysfunction, along with lower levels of abuse compared with included participants (eTable 2 in Supplement 1). As shown in eTable 3 in Supplement 1, the prevalence of PSU among Chinese adolescents was found to be 35.4% (95% CI, 30.0%-41.2%). Additionally, the prevalence of each ACE domain ranged from 8.1% (95% CI, 7.4%-8.8%) for abuse to 27.2% (95% CI, 25.0%-29.6%) for household dysfunction. Nearly 96.0% of participants reported exposure to at least 1 ACE, with 6.3% experiencing 4 or more ACEs.

**Table 1.** Characteristics of Study Participants Across Sexual Orientation Categories

Characteristics	Participants, No. (%)^a^					*P* value^b^
	Overall (N = 85 0460)	Heterosexual (n = 56 945)	Homosexual (n = 1484)	Bisexual (n = 6429)	Not sure (n = 4428)	
Age, mean (SD), y	14.92 (1.77)	15.22 (1.71)	14.77 (1.75)	14.84 (1.69)	14.18 (1.69)	<.001
Sex						
Male	42 414 (49.9)	30 971 (54.4)	585 (39.4)	1146 (17.8)	1785 (40.3)	<.001
Female	42 632 (50.1)	25 974 (45.6)	899 (60.6)	5283 (82.2)	2643 (59.7)	
Ethnicity						
Han	70 157 (83.2)	47 107 (83.3)	1218 (83.2)	5346 (83.8)	3562 (81.5)	.01
Others^c^	14 208 (16.8)	9441 (16.7)	246 (16.8)	1030 (16.2)	809 (18.5)	
Place of residence						
Urban	39 182 (46.5)	25 369 (44.9)	854 (58.0)	3742 (58.7)	1956 (44.7)	<.001
Rural	45 156 (53.5)	31 162 (55.1)	619 (42.0)	2637 (41.3)	2420 (55.3)	
PSU						
No	55 855 (66.1)	36 106 (63.7)	909 (61.8)	4018 (62.7)	3223 (73.2)	<.001
Yes	28 704 (33.9)	20 584 (36.3)	563 (38.2)	2387 (37.3)	1182 (26.8)	
ACEs						
Abuse						
Sexual abuse						
No	82 851 (97.7)	55 660 (97.9)	1379 (93.2)	6083 (94.7)	4358 (98.6)	<.001
Yes	1978 (2.3)	1217 (2.1)	100 (6.8)	338 (5.3)	63 (1.4)	
Physical abuse						
No	82 785 (97.6)	55 658 (97.9)	1395 (94.3)	6129 (95.4)	4336 (98.1)	<.001
Yes	2043 (2.4)	1216 (2.1)	84 (5.7)	294 (4.6)	85 (1.9)	
Emotional abuse						
No	80 055 (94.4)	54 198 (95.3)	1289 (87.2)	5660 (88.2)	4220 (95.5)	<.001
Yes	4745 (5.6)	2682 (4.7)	190 (12.8)	760 (11.8)	201 (4.5)	
Neglect						
Physical neglect						
No	78 871 (93.1)	53 154 (93.5)	1274 (86.3)	5785 (90.2)	4151 (94.0)	<.001
Yes	5853 (6.9)	3693 (6.5)	203 (13.7)	630 (9.8)	263 (6.0)	
Emotional neglect						
No	74 359 (87.7)	50 867 (89.4)	1157 (78.2)	5387 (83.9)	3747 (84.9)	<.001
Yes	10 436 (12.3)	6014 (10.6)	322 (21.8)	1034 (16.1)	668 (15.1)	
Household dysfunction						
Parental separation or divorce						
No	72 859 (88.7)	49 206 (88.9)	1190 (83.6)	5265 (83.7)	3805 (90.4)	<.001
Yes	9258 (11.3)	6121 (11.1)	234 (16.4)	1029 (16.3)	406 (9.6)	
Household criminality						
No	80 972 (98.6)	54 524 (98.6)	1374 (96.6)	6177 (98.1)	4173 (99.1)	<.001
Yes	1135 (1.4)	794 (1.4)	49 (3.4)	117 (1.9)	38 (0.9)	
Household domestic violence						
No	74 634 (90.9)	50 425 (91.1)	1193 (83.8)	5364 (85.2)	3875 (92.0)	<.001
Yes	7479 (9.1)	4898 (8.9)	231 (16.2)	930 (14.8)	336 (8.0)	
Household mental illness						
No	81 136 (98.8)	54 760 (99.0)	1377 (96.8)	6103 (97.0)	4167 (99.0)	<.001
Yes	972 (1.2)	559 (1.0)	45 (3.2)	191 (3.0)	44 (1.0)	
Household substance abuse						
No	81 270 (99.0)	54 798 (99.1)	1381 (97.0)	6163 (97.9)	4176 (99.2)	<.001
Yes	838 (1.0)	521 (0.9)	42 (3.0)	131 (2.1)	35 (0.8)	
Family financial problems						
No	77 600 (94.5)	52 306 (94.5)	1324 (93.1)	5909 (93.9)	3978 (94.5)	.02
Yes	4509 (5.5)	3015 (5.5)	98 (6.9)	385 (6.1)	233 (5.5)	
Death of parents						
No	80 129 (97.6)	53 951 (97.5)	1378 (96.9)	6132 (97.4)	4124 (97.9)	.14
Yes	1979 (2.4)	1369 (2.5)	44 (3.1)	162 (2.6)	87 (2.1)	
Witness of community violence						
No	76 797 (93.5)	51 764 (93.6)	1276 (89.7)	5609 (89.1)	4004 (95.1)	<.001
Yes	5317 (6.5)	3561 (6.4)	147 (10.3)	685 (10.9)	207 (4.9)	
Sex discrimination						
No	79 084 (96.3)	53 647 (97.0)	1263 (88.8)	5721 (90.9)	4057 (96.3)	<.001
Yes	3026 (3.7)	1675 (3.0)	159 (11.2)	573 (9.1)	154 (3.7)	
No. of ACEs						
0	50 252 (61.5)	34 641 (62.8)	622 (44.0)	3004 (47.9)	2669 (63.6)	<.001
1	17 667 (21.6)	11 848 (21.5)	341 (24.1)	1472 (23.5)	894 (21.3)	
2	7343 (9.0)	4801 (8.7)	178 (12.6)	823 (13.1)	339 (8.1)	
3	3257 (4.0)	2012 (3.6)	112 (7.9)	456 (7.3)	155 (3.7)	
≥4	3228 (3.9)	1871 (3.4)	162 (11.4)	520 (8.3)	139 (3.3)	

Abbreviations: ACEs, adverse childhood experiences; PSU, problematic smartphone use.

^a^ Missing data: 681 for ethnicity, 708 for place of residence, 487 for PSU, 216 for sexual abuse, 219 for physical abuse, 248 for emotional abuse, 325 for physical neglect, 328 for emotional neglect, 2929 for parental separation or divorce, 2939 for household criminality, 2933 for household domestic violence, 2938 for household mental illness, 2938 for household substance abuse, 2937 for family financial problems, 2938 for death of parents, 2932 for witness of community violence, 2936 for sex discrimination, 3299 for ACE scores, and 15 960 for sexual orientation.

^b^ To compare characteristics across different sexual orientation and gender expression groups, analysis of variance was used for continuous variables and χ^2^ tests were applied for categorical variables.

^c^ Others refers to Miao, Hui, Yi, Dai, and other ethnic groups.

### Associations Between Sexual Orientation and Gender Expression and ACEs

In Table 2, the adjusted linear regression models revealed that nonheterosexual adolescents, compared with their heterosexual counterparts, were more likely to report a higher number of ACEs (β = 0.19; 95% CI, 0.13-0.26). Individuals with moderate or high GNC exhibited a greater likelihood of reporting a higher number of ACEs (β = 0.12; 95% CI, 0.09-0.15) in comparison with those with low GNC.

**Table 2.** The Total Number of Adverse Childhood Experiences (ACEs) Across Sexual Orientations and Gender Expressions

Characteristic	Total No. of ACEs, β (95% CI)^a^
Sexual orientation	
Heterosexual	1 [Reference]
Nonheterosexual	0.19 (0.13-0.26)
Gender nonconformity	
Low GNC	1 [Reference]
Moderate or high GNC	0.12 (0.09-0.15)

^a^ Models were adjusted for age, sex, ethnicity, and place of residence.

Further analysis of individual ACE components, as demonstrated in the weighted logistic regression models, showed that homosexual individuals had the highest adjusted associations with sexual abuse (adjusted odds ratio [aOR], 7.27; 95% CI, 4.35-11.55), followed by household substance abuse (aOR, 4.73; 95% CI, 2.38-8.51) and sex discrimination (aOR, 4.14; 95% CI, 3.03-5.56). Similar results were observed among bisexual individuals and those with high GNC (see eTable 4 in Supplement 1).

### Associations Between ACEs and PSU

In the adjusted Poisson regression models, adolescents who reported at least 2 ACEs showed a heightened likelihood of PSU (eg, 2 ACEs: adjusted prevalence ratio [APR], 1.25; 95% CI, 1.18-1.33). Specifically, individuals who reported experiencing abuse demonstrated a significantly higher prevalence of PSU compared with those who did not report such experiences (sexual abuse APR, 1.51; 95% CI, 1.39-1.64; physical abuse APR, 1.44; 95% CI, 1.30-1.59; emotional abuse APR, 1.57; 95% CI, 1.42-1.73) (Table 3).

**Table 3.** Associations Between Adverse Childhood Experiences (ACEs) and Problematic Smartphone Use (PSU) Among Study Participants^a^^,^^b^

ACEs	Participants, No. (%)		PR (95% CI)	APR (95% CI)^c^
	Non-PSU	PSU		
No. of ACEs				
0	2478 (4.6)	1137 (4.1)	1 [Reference]	1 [Reference]
1	34 267 (63.9)	13 541 (48.7)	0.92 (0.83-1.02)	0.93 (0.84-1.03)
2	10 730 (20.0)	7095 (25.5)	1.27 (1.22-1.33)	1.25 (1.18-1.33)
3	3628 (6.8)	3274 (11.8)	1.49 (1.39-1.59)	1.45 (1.34-1.57)
≥4	2502 (4.7)	2733 (9.8)	1.61 (1.51-1.72)	1.56 (1.47-1.66)
Each ACE component				
Abuse				
Sexual abuse				
No	54 859 (98.4)	27 569 (96.2)	1 [Reference]	1 [Reference]
Yes	889 (1.6)	1078 (3.8)	1.56 (1.42-1.72)	1.51 (1.39-1.64)
Physical abuse				
No	54 725 (98.2)	27 640 (96.5)	1 [Reference]	1 [Reference]
Yes	1024 (1.8)	1005 (3.5)	1.42 (1.29-1.57)	1.44 (1.30-1.59)
Emotional abuse				
No	53 389 (95.8)	26 283 (91.8)	1 [Reference]	1 [Reference]
Yes	2353 (4.2)	2356 (8.2)	1.46 (1.34-1.60)	1.57 (1.42-1.73)
Neglect				
Physical neglect				
No	52 690 (94.6)	25 814 (90.2)	1 [Reference]	1 [Reference]
Yes	3002 (5.4)	2803 (9.8)	1.43 (1.31-1.56)	1.43 (1.31-1.56)
Emotional neglect				
No	49 442 (88.7)	24 563 (85.8)	1 [Reference]	1 [Reference]
Yes	6298 (11.3)	4070 (14.2)	1.16 (1.07-1.26)	1.18 (1.08-1.29)
Household dysfunction				
Parental separation or divorce				
No	50 039 (93.0)	24 232 (86.8)	1 [Reference]	1 [Reference]
Yes	3760 (7.0)	3669 (13.2)	1.15 (1.06-1.25)	1.17 (1.11-1.22)
Household criminality				
No	53 260 (99.0)	27 470 (98.5)	1 [Reference]	1 [Reference]
Yes	536 (1.0)	429 (1.5)	1.33 (1.21-1.46)	1.31 (1.20-1.43)
Household domestic violence				
No	53 388 (99.2)	27 475 (98.5)	1 [Reference]	1 [Reference]
Yes	407 (0.8)	425 (1.5)	1.48 (1.36-1.61)	1.44 (1.35-1.54)
Household mental illness				
No	51 558 (95.8)	25 659 (92.0)	1 [Reference]	1 [Reference]
Yes	2238 (4.2)	2241 (8.0)	1.31 (1.17-1.46)	1.29 (1.14-1.45)
Household substance abuse				
No	52 638 (97.8)	27 094 (97.1)	1 [Reference]	1 [Reference]
Yes	1157 (2.2)	806 (2.9)	1.44 (1.22-1.70)	1.39 (1.21-1.58)
Family financial problems				
No	51 558 (95.8)	25 659 (92.0)	1 [Reference]	1 [Reference]
Yes	2238 (4.2)	2241 (8.0)	1.43 (1.27-1.62)	1.28 (1.21-1.37)
Death of parent				
No	52 638 (97.8)	27 094 (97.1)	1 [Reference]	1 [Reference]
Yes	1157 (2.2)	806 (2.9)	1.18 (1.06-1.31)	1.11 (1.03-1.19)
Witness of community violence				
No	51 077 (94.9)	25 335 (90.8)	1 [Reference]	1 [Reference]
Yes	2721 (5.1)	2568 (9.2)	1.42 (1.31-1.53)	1.33 (1.28-1.38)
Sex discrimination				
No	52 202 (97.0)	26 484 (94.9)	1 [Reference]	1 [Reference]
Yes	1596 (3.0)	1415 (5.1)	1.35 (1.24-1.47)	1.38 (1.26-1.50)

Abbreviations: APR, adjusted prevalence ratio; PR, prevalence ratio.

^a^ PSU was measured by the Smartphone Addiction Scale-Short Version (SAS-SV) scores, ranging from 10 to 60; male students with SAS-SV scores 31 and higher and female students with SAS-SV scores 33 and higher were classified as the PSU group.

^b^ Models showed good model fit, with the Wald test reporting *P* < .001 and the Pearson goodness-of-fit test reporting *P* > .10.

^c^ Models were adjusted for age, sex, ethnicity, and place of residence.

### Associations Between ACEs and PSU Across Sexual Orientation and Gender Expression Categories

In the fully adjusted Poisson model, we found no significant multiplicative interaction was observed between ACEs and sexual orientation on PSU (APR, 1.00; 95% CI, 0.99-1.02; *P* = .78). However, a significant interaction was detected between ACEs and GNC (APR, 0.98; 95% CI, 0.97-0.99; *P* = .03) (eTable 5 in Supplement 1). Regarding sexual orientations, individuals identifying as homosexual or bisexual and reporting at least 2 ACEs exhibited an increased prevalence of PSU. Specifically, among homosexual students, adolescents who experienced 4 or more ACEs showed a significantly increased prevalence of PSU (APR, 1.79; 95% CI, 1.64-1.96). Similarly, a markedly higher prevalence of PSU was observed among bisexual individuals with 4 or more ACEs (APR, 1.60; 95% CI, 1.41-1.80). Concerning gender expression categories, a significantly higher prevalence of PSU was noticed among moderate or high GNC adolescents with 4 or more ACEs (eg, high GNC adolescents APR, 1.78; 95% CI, 1.60-1.98) compared with low GNC adolescents without ACEs (Table 4).

**Table 4.** Associations Between the Number of Adverse Childhood Experiences (ACEs) and Problematic Smartphone Use (PSU) Among Different Sexual Orientations and Gender Expressions^a^

No. of ACEs	PSU APR (95% CI)^b^^,^^c^					
	Sexual orientation^d^			Gender nonconformity^e^		
	Heterosexual	Homosexual	Bisexual	Low	Moderate	High
0	1 [Reference]	0.88 (0.64-1.19)	1.20 (0.91-1.57)	1 [Reference]	1.11 (0.99-1.24)	1.14 (1.01-1.28)
1	0.94 (0.85-1.03)	1.05 (0.83-1.33)	1.02 (0.94-1.12)	0.95 (0.86-1.04)	0.99 (0.90-1.10)	1.13 (0.98-1.32)
2	1.25 (1.17-1.25)	1.31 (1.07-1.62)	1.25 (1.10-1.43)	1.26 (1.20-1.33)	1.36 (1.25-1.48)	1.47 (1.34-1.62)
3	1.44 (1.35-1.54)	1.47 (1.27-1.69)	1.55 (1.36-1.76)	1.48 (1.38-1.58)	1.58 (1.46-1.72)	1.46 (1.31-1.64)
≥4	1.54 (1.41-1.67)	1.79 (1.64-1.96)	1.60 (1.41-1.80)	1.59 (1.49-1.69)	1.66 (1.53-1.80)	1.78 (1.60-1.98)

Abbreviation: APR, adjusted prevalence ratio.

^a^ Models showed good model fit, with the Wald test reporting *P* < .001 and the Pearson goodness-of-fit test reporting *P* > .10.

^b^ PSU was measured by the Smartphone Addiction Scale-Short Version (SAS-SV) scores, ranging from 10 to 60; male students with SAS-SV scores 31 or higher and female students with SAS-SV scores 33 or higher were classified as the PSU group.

^c^ The models for APRs were adjusted for age, sex, ethnicity, and place of residence.

^d^ PRs were calculated for associations of the number of ACEs categories (0, 1, 2, 3, ≥4) with the studied PSU outcome across sexual orientation categories using heterosexual students with an ACE score = 0 as reference.

^e^ PRs were calculated for associations of the number of ACE categories (0, 1, 2, 3, ≥4) with the studied PSU outcome across gender nonconformity categories, using adolescents with low GNC and an ACE score = 0 as reference.

Table 5 illustrates that experiencing any 3 ACE categories (abuse, neglect, and household dysfunction) was associated with an increased prevalence of PSU across different sexual orientation and gender expression subgroups. For example, among homosexual and bisexual students, those with a history of abuse demonstrated markedly elevated prevalence of PSU (homosexual students APR, 1.66; 95% CI, 1.46-1.88; bisexual students APR, 1.57; 95% CI, 1.36-1.81), followed by household dysfunction (homosexual students APR, 1.53; 95% CI, 1.40-1.68; bisexual students APR, 1.41; 95% CI, 1.24-1.59) and neglect (homosexual students APR, 1.35; 95% CI, 1.07-1.70; bisexual students APR, 1.37; 95% CI, 1.20-1.58). Similar results were observed among individuals with moderate or high GNC.

**Table 5.** Associations Between Categorized Adverse Childhood Experiences (ACEs) and Problematic Smartphone Use (PSU) Among Different Sexual Orientations and Gender Expressions^a^

ACE category	PSU APR (95% CI)^b^^,^^c^					
	Sexual orientation^d^			Gender nonconformity^e^		
	Heterosexual	Homosexual	Bisexual	Low	Moderate	High
Abuse						
ACE not reported	1 [Reference]	1.09 (0.94-1.25)	1.09 (1.01-1.18)	1 [Reference]	1.08 (1.07-1.11)	1.20 (1.13-1.27)
ACE reported	1.52 (1.39-1.67)	1.66 (1.46-1.88)	1.57 (1.36-1.81)	1.56 (1.43-1.70)	1.64 (1.47-1.85)	1.59 (1.43-1.75)
Neglect						
ACE not reported	1 [Reference]	1.13 (0.98-1.30)	1.11 (1.04-1.19)	1 [Reference]	1.14 (1.07-1.21)	1.18 (1.05-1.33)
ACE reported	1.25 (1.16-1.35)	1.35 (1.07-1.70)	1.37 (1.20-1.58)	0.95 (0.89-1.01)	1.02 (0.97-1.24)	1.14 (1.05-1.24)
Household dysfunction						
ACE not reported	1 [Reference]	1.08 (0.86-1.35)	1.12 (1.06-1.18)	1 [Reference]	1.07 (1.03-1.11)	1.19 (1.11-1.28)
ACE reported	1.33 (1.28-1.39)	1.53 (1.40-1.68)	1.41 (1.24-1.59)	1.35 (1.29-1.42)	1.45 (1.34-1.55)	1.57 (1.45-1.70)

Abbreviation: APR, adjusted prevalence ratio.

^a^ Models showed good model fit, with the Wald test reporting *P* < .001 and the Pearson goodness-of-fit test reporting *P* > .10.

^b^ PSU was measured by the Smartphone Addiction Scale-Short Version (SAS-SV) scores, ranging from 10 to 60; male students with SAS-SV scores 31 or higher and female students with SAS-SV scores 33 or higher were classified as the PSU group.

^c^ The models for APRs were adjusted for age, sex, ethnicity, and place of residence.

^d^ PRs were calculated for associations of each of the 3 ACE categories (abuse, neglect, and household dysfunction) with the studied PSU outcome across sexual orientation categories, using heterosexual students not reporting the ACE category in question as reference.

^e^ PRs were calculated for associations of each of the 3 ACE categories (abuse, neglect, and household dysfunction) with the studied PSU outcome across gender nonconformity categories, using adolescents with low GNC and an ACE score = 0 as reference.

## Discussion

This cross-sectional study is one of the first to explore the associations between ACEs and PSU across different subgroups of sexual orientation and gender expression among school-based Chinese adolescents. The findings highlight a range of prevalence rates for individual ACE components, with rates varying from 8.1% for abuse to 27.2% for household dysfunction. Notably, nearly 96.0% of participants reported exposure to at least 1 ACE, with 6.3% experiencing 4 or more ACEs. Consistent with prior research,^17,18,31^ our results indicate that homosexual adolescents or those with high GNC were more inclined to report ACEs compared with their heterosexual or low GNC counterparts. These results may stem from the mistreatment experienced by sexual minority individuals, which can be attributed to heterosexism, a set of attitudes that favor heterosexuality while devaluing other sexual orientations. Moreover, the adverse social context faced by these populations contributes to their elevated ACE prevalence. Given their deviation from traditional social norms in terms of sexual orientation and gender expression, they often encounter marginalization, stigma, and discrimination.^32,33^

Furthermore, we observed a consistent positive association between ACEs and PSU, which aligns with prior research findings.^34,35^ Drawing upon the compensatory internet use theory, individuals may engage in excessive smartphone usage as a coping mechanism to alleviate stressors.^5,6^ ACEs represent a significant source of negative stress that can progressively impair the stress response system, including the processes governing the intensity and duration of stress responses, as well as neurological structures and reward system-related functioning.^36,37^ This impairment renders adolescents more susceptible to maladaptive coping strategies and addictive behaviors, such as PSU.^38^ Within the context of a vulnerable population with ACEs, the widespread availability of smartphones and access to online features offer avenues for temporary distraction and relief from adverse real-life environments and negative emotions, potentially contributing to increased levels of excessive smartphone use.^39^ Moreover, the ACE experiences may detrimentally affect adolescents’ self-perception, potentially driving PSU as a means to cultivate positive beliefs and enhance their self-image.^40,41,42^

In addition, our study revealed that adolescents exposed to ACEs exhibited an elevated susceptibility to PSU, regardless of their sexual orientations or gender expressions. We also identified significant associations between combinations of ACEs and both nonheterosexual orientation or high GNC and PSU. Specifically, a higher prevalence of PSU was evident when higher ACE scores or each of the 3 categorized ACEs (ie, abuse, neglect, and household dysfunction) were combined with either a nonheterosexual orientation or a high GNC identity. While prior research has not extensively explored the association between ACEs and PSU across different sexual orientations and gender expressions, existing literature does report associations of ACEs with other mental health and behavioral issues among diverse sexual orientations. Studies conducted in the US among adults and high school students have consistently reported that nonheterosexual individuals face an elevated prevalence of co-occurring substance use and mental health problems and are more likely to be exposed to a higher number of ACEs.^43,44^ These findings can be contextualized within the framework of the minority stress model, which posits that nonheterosexual and GNC individuals often encounter various minority stressors (eg, prejudice, discrimination, harassment, and violence) in their daily lives. Consequently, sexual minority and GNC youths may resort to PSU as a maladaptive coping mechanism in response to experiences of social and minority stress.^15,16^ Moreover, ACEs could also be perceived as traumatic stressors associated with PSU^10^; when combined with nonheterosexual orientation or GNC, their deleterious cumulative influences could be stronger. These findings highlight the significance of preventing ACEs for adolescents, particularly with a targeted approach toward nonheterosexual adolescents and those with high GNC identities.

### Limitations

There are several limitations to this study. First, the cross-sectional nature of our data restricts our ability to establish definitive causal relationships between ACEs and PSU among different sexual orientation and gender expression groups. Second, our study focused on Chinese middle and high school students, which may limit the generalizability of findings to populations less reliant on smartphones, such as adolescents from cultures with lower smartphone usage rates. Third, it is essential to acknowledge potential biases arising from differential reporting probabilities for childhood adversities and PSU across the compared groups. Previous research^8^ indicates that nonheterosexual individuals are more likely to recall and report ACEs, potentially introducing information bias. Fourth, the data collection method used in schools may have excluded nonheterosexual students or individuals with high GNC who do not attend school, possibly omitting those experiencing more severe PSU issues than those captured in the school-based sample. Fifth, the timing of our study amid the COVID-19 pandemic, characterized by increased reliance on electronic devices, especially among adolescents engaged in online learning, may have influenced the prevalence of PSU. This context could complicate the interpretation of associations with ACEs.^45,46^ However, it is noteworthy that our survey was conducted within schools where offline teaching had resumed, mitigating some of the confounding effects.

## Conclusions

In this cross-sectional study of nationally representative school-based participants, we found that sexual minorities and gender-nonconforming individuals were more likely to experience ACEs. Adolescents exposed to ACEs demonstrated a heightened susceptibility to PSU, irrespective of their sexual orientations or gender expressions. Furthermore, the combination of higher ACE scores with either a nonheterosexual orientation or a gender-nonconforming identity significantly increases the prevalence of PSU. Therefore, the findings suggest that preventing ACEs may be beneficial in mitigating PSU among adolescents, particularly for nonheterosexual adolescents and those with high levels of GNC. However, future randomized clinical trials are needed to confirm these conclusions.

## Notes

Supplement 1.
eMethods.
**eFigure.** Directed Acyclic Graph (DAG) for Adverse Childhood Experiences (ACEs), Problematic Smartphone Use (PSU), and Other Covariates Across Sexual Orientation and Gender Expression Groups **eTable 1. **Characteristics of Study Participants Across Gender Expressions **eTable 2. **Characteristics of the Included and Excluded Participants **eTable 3. **Prevalence Estimates of PSU and 95% CIs by Study Characteristics **eTable 4. **Categorized ACEs Across Sexual Orientation and Gender Expressions **eTable 5. **Interactions Between ACE and Sexual Orientation/Gender Expression on PSU
eReferences

Supplement 2.
Data Sharing Statement
