# Supplementary material for: Childhood Adversity and Adolescent Smartphone Use Across Sexual Orientation and Gender Expression
Source: JAMA Netw Open. 2024 Apr 12;7(4):e246448. doi: 10.1001/jamanetworkopen.2024.6448 (PMC11015349; doi:10.1001/jamanetworkopen.2024.6448)
Supplement: Supplement 1. — eMethods. eFigure. Directed Acyclic Graph (DAG) for Adverse Childhood Experiences (ACEs), Problematic Smartphone Use (PSU), and Other Covariates Across Sexual Orientation and Gender Expression Groups eTable 1. Characteristics of Study Participants Across Gender Expressions eTable 2. Characteristics of the Included and Excluded Participants eTable 3. Prevalence Estimates of PSU and 95% CIs by Study Characteristics eTable 4. Categorized ACEs Across Sexual Orientation and Gender Expressions eTable 5. Interactions Between ACE and Sexual Orientation/Gender Expression on PSU eReferences [file jamanetwopen-e246448-s001.pdf]

Supplemental Online Content

Zheng X, Jiang W, Peng S, et al. Childhood adversity and adolescent smartphone use across sexual orientation and gender expression. *JAMA Netw Open*. 2024;7(4):e246448. doi:10.1001/jamanetworkopen.2024.6448

eMethods.
eFigure. Directed Acyclic Graph (DAG) for Adverse Childhood Experiences (ACEs), Problematic Smartphone Use (PSU), and Other Covariates Across Sexual Orientation and Gender Expression Groups
eTable 1. Characteristics of Study Participants Across Gender Expressions
eTable 2. Characteristics of the Included and Excluded Participants
eTable 3. Prevalence Estimates of PSU and 95% CIs by Study Characteristics
eTable 4. Categorized ACEs Across Sexual Orientation and Gender Expressions
eTable 5. Interactions Between ACE and Sexual Orientation/Gender Expression on PSU
eReferences

This supplemental material has been provided by the authors to give readers additional information about their work.

© 2024 Zheng X et al. *JAMA Network Open.*

eMethods.

Study design and population

The current study utilized data from the 2021 School-based Chinese Adolescents Health Survey (SCAHS), an ongoing survey of health-related behaviors among Chinese adolescents (grades 7-12) [1]. SCAHS collects large-scale cross-sectional data (every two years since 2007) and longitudinal data (between 2009 and 2012) through self-reported questionnaires administered in classrooms [2]. The 2021 SCAHS utilized a multi-stage, stratified cluster, random sampling method, and the procedures for data collection were as follows. In stage 1, we divided Chinese provinces into three economic strata (high-economic level, middle-economic level, low-economic level) according to per capita gross domestic product (GDP) level. Based on the proportion of each stratum and cooperation, eight provinces were selected to participate in our study: Guangdong, Henan, and Shandong (high-level); Chongqing and Yunnan (middle-level); Guizhou, Liaoning, and Heilongjiang (low-level). In each province, three cities were randomly selected with the assistance of local education bureaus. In stage 2, six junior high schools, four senior high schools, and two vocational high schools were randomly selected based on the overall proportion of the three types of schools in the chosen cities. In stage 3, two classes were randomly selected from each grade within the chosen schools. The SCAHS achieved a high response rate due to the full endorsement received from local education authorities. The randomly selected schools and classes achieved a response rate of 100% in this survey. All available students in the selected classes except those who suffered from severe mental or physical disorders, as identified by the head

© 2024 Zheng X et al. *JAMA Network Open.*

teacher and/or health care physicians, were invited to participate in the study. We ensured that a detailed written informed consent was provided to both students and their legal guardians. This document outlined the risks, benefits, and procedures associated with the survey, ensuring full understanding prior to participation. The survey was conducted by trained investigators available to address any queries or confusion the participants might have had regarding the structured questionnaire. This ensured clarity and consistency in responses. Participants completed the survey within a single session at school after being informed of the study's objectives and procedures. All participants were expected to complete the anonymous questionnaire within 45 minutes independently without the presence of teachers. This approach emphasized anonymity as a means to minimize potential external influences on students' responses. Furthermore, the investigators reviewed the completeness of the questionnaires before the participants left the site to ensure the accuracy and reliability of the data collected.

ACEs

The simplified version of the Childhood Trauma Questionnaire (CTQ-SF) [3], a 28-item self-report questionnaire, was employed to assess the history of abuse and neglect that the participants had experienced or are experiencing. It consists of five subscales, including sexual abuse, physical abuse, emotional abuse, physical neglect, and emotional neglect. Respondents selected options on a five-point Likert scale ranging from "never" to "very often" with statements such as "People in my family hit me so hard that it left me with bruises and marks," "People in my family said hurtful or

© 2024 Zheng X et al. *JAMA Network Open.*

insulting things to me." The sum of the items provides an overall CTQ-SF score, with a higher score indicating a more severe ACEs. According to the cut-off scores for CTQ-SF subscales (emotional abuse score ≥ 13, physical abuse score ≥ 10, sexual abuse score ≥ 8, emotional neglect score ≥ 15, and physical neglect score ≥ 10) [4]. We calculated the total CTQ-SF scores across items and the CTQ-SF scores in each subscale. Adolescents were classified according to whether they experienced childhood trauma history in respective categories accordingly. CTQ-SF was widely used in Chinese adolescents and proved to have good reliability and validity[5,6]. The CTQ-SF exhibited high internal consistency in the present study, with a Cronbach's α of 0.89.

Household dysfunction was asked with a single item adapted from the original ACE study[7]. Questions asked respondents whether they have the following experiences during their lives: parental separation or divorce, household criminality, household domestic violence, household mental illness, household substance abuse, family financial problems, death of parent, witness of community violence, and sex discrimination, and the response were coded "No"=0 "Yes"=1.

Sexual orientation and gender expression

Sexual orientation was assessed using the question "Which gender do you think you are romantically attracted to?" with response options including male, female, both male and female, not sure, unwilling to answer, and neither men nor women[8]. Students were categorized as "heterosexual", "homosexual", "bisexual", and "not sure" based on their response and biological sex. Adolescents who chose "unwilling to answer" (n=13583,

© 2024 Zheng X et al. *JAMA Network Open.*

15.9%) and "neither men nor women" (n=1314, n=1.5%) were excluded from further association statistical analysis.

Gender expression was measured with a validated measure by asking the following question: "A person's appearance, style, dress, or the way they walk or talk may affect how people describe them. How do you think people at school would describe you?"[9] The response options were: "very feminine," "mostly feminine," "somewhat feminine," "equally feminine and masculine," "somewhat masculine," "mostly masculine," and "very masculine." Based on a student's response to this and to the question "What is your sex?" (response options were "female" or "male") Students were categorized from most gender conforming (indicating very feminine female students and very masculine male students) to most gender nonconforming (indicating very masculine female students and very feminine male students) [10]. In this study, because of the limited number of mostly or very masculine female students and the limited number of mostly or very feminine male students, we adopted a 3-level GNC variable based on previous studies, including one of our previous works [11,12]: (1) high GNC (somewhat, mostly, and very masculine female students and somewhat, mostly, and very feminine male students), (2) moderate GNC (equally feminine and masculine female and male students), and (3) low GNC (somewhat, mostly, and very feminine female students and somewhat, mostly, and very masculine male students).

Ethnicity

Ask the participants "if their ethnicity is Han?" and if not, ask them to indicate their

© 2024 Zheng X et al. *JAMA Network Open.*

specific ethnicity. Ethnicity was categorized into two groups: Han and ethnic minority, with the Han ethnicity representing the predominant group in China numerically.

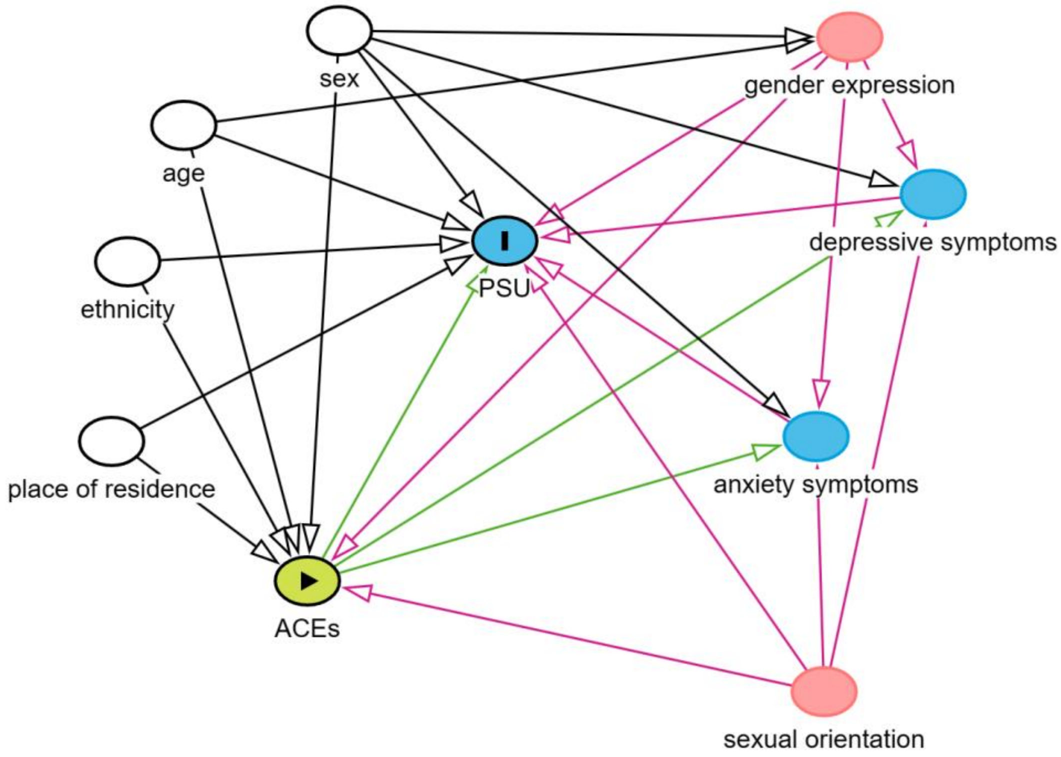

eFigure. Directed Acyclic Graph (DAG) for Adverse Childhood Experiences (ACEs), Problematic Smartphone Use (PSU), and Other Covariates Across Sexual Orientation and Gender Expression Groups

[The minimally sufficient adjustment set was determined using the DAGitty software. The simplified DAG was generated by the online resource DAGitty (http://www.dagitty.net/dags.html#)].

© 2024 Zheng X et al. *JAMA Network Open.*

eTable 1. Characteristics of Study Participants Across Gender Expressions

Characteristics	Gender Nonconformity[a]				P-value*
	Overall, n (%)	Low, n (%)	Moderate, n (%)	High, n (%)	
**Total**	85046 (100)	56471 (67.6)	22160 (26.5)	4901 (5.9)	
**Age, mean (SD)**	14.92 (1.77)	15.05 (1.76)	14.66 (1.75)	14.63 (1.77)	<0.001
**Sex**					
Male	42414 (49.9)	31297 (55.4)	8350 (37.7)	1899 (38.7)	
Female	42632 (50.1)	25174 (44.6)	13810 (62.3)	3002 (61.3)	<0.001
**Ethnicity[a]**					
Han	70157 (83.2)	47095 (84.0)	17860 (81.4)	4019 (82.8)	
Others[b]	14208 (16.8)	8992 (16.0)	4079 (18.6)	834 (17.2)	<0.001
**Place of residence[a]**					
Urban	39182 (46.5)	26341 (47.0)	10042 (45.7)	2181 (44.9)	
Rural	45156 (53.5)	29694 (53.0)	11920 (54.3)	2673 (55.1)	<0.001
**PSU[a]**					
No	55855 (66.1)	37391 (66.5)	14451 (65.6)	2996 (61.5)	
Yes	28704 (33.9)	18828 (33.5)	7593 (34.4)	1873 (38.5)	<0.001
**ACEs**					
**Abuse**					
**Sexual abuse[a]**					
No	82851 (97.7)	55236 (97.9)	21590 (97.6)	4643 (94.9)	
Yes	1978 (2.3)	1169 (2.1)	531 (2.4)	247 (5.1)	<0.001
**Physical abuse[a]**					
No	82785 (97.6)	55260 (98.0)	21493 (97.2)	4649 (95.1)	
Yes	2043 (2.4)	1145 (2.0)	628 (2.8)	240 (4.9)	<0.001
**Emotional abuse[a]**					

© 2024 Zheng X et al. *JAMA Network Open.*

eTable 1. Characteristics of study participants across gender expressions (continued)

No	80055 (94.4)	53935 (95.6)	20535 (92.8)	4292 (87.7)	
Yes	4745 (5.6)	2467 (4.4)	1596 (7.2)	601 (12.3)	<0.001
**Neglect**					
**Physical neglect**[a]					
No	78871 (93.1)	52999 (94.0)	20308 (91.9)	4276 (87.6)	
Yes	5853 (6.9)	3376 (6.0)	1796 (8.1)	608 (12.4)	<0.001
**Emotional neglect**[a]					
No	74359 (87.7)	50648 (89.8)	18716 (84.6)	3890 (79.6)	
Yes	10436 (12.3)	5755 (10.2)	3409 (15.4)	1000 (20.4)	<0.001
**Household dysfunction**					
**Parental separation or divorce**[a]					
No	72859 (88.7)	48863 (89.3)	18844 (88.0)	4002 (85.2)	
Yes	9258 (11.3)	5882 (10.7)	2562 (12.0)	697 (14.8)	<0.001
**Household criminality**[a]					
No	80972 (98.6)	54042 (98.7)	21070 (98.4)	4606 (98.0)	
Yes	1135 (1.4)	696 (1.3)	334 (1.6)	92 (2.0)	<0.001
**Household domestic violence**[a]					
No	74634 (90.9)	50264 (91.8)	19179 (89.6)	4010 (85.4)	
Yes	7479 (9.1)	4478 (8.2)	2227 (10.4)	688 (14.6)	<0.001
**Household mental illness**[a]					
No	81136 (98.8)	54201 (99.0)	21084 (98.5)	4593 (97.8)	
Yes	972 (1.2)	536 (1.0)	322 (1.5)	105 (2.2)	<0.001
**Household substance abuse**[a]					
No	81270 (99.0)	54267 (99.1)	21154 (98.8)	4589 (97.7)	
Yes	838 (1.0)	470 (0.9)	253 (1.2)	109 (2.3)	<0.001

© 2024 Zheng X et al. *JAMA Network Open.*

eTable 1. Characteristics of study participants across gender expressions (continued)

**Family financial problems[a]**					
No	77600 (94.5)	52020 (95.0)	20044 (93.6)	4336 (92.3)	
Yes	4509 (5.5)	2718 (5.0)	1363 (6.4)	362 (7.7)	<0.001
**Death of parents[a]**					
No	80129 (97.6)	53469 (97.7)	20851 (97.4)	4567 (97.2)	
Yes	1979 (2.4)	1269 (2.3)	555 (2.6)	131 (2.8)	0.02
**Witness of community violence[a]**					
No	76797 (93.5)	51484 (94.0)	19887 (92.9)	4222 (89.9)	
Yes	5317 (6.5)	3259 (6.0)	1520 (7.1)	476 (10.1)	<0.001
**Sex discrimination[a]**					
No	79084 (96.3)	53159 (97.1)	20403 (95.3)	4291 (91.3)	
Yes	3026 (3.7)	1581 (2.9)	1003 (4.7)	407 (8.7)	<0.001
**Number of ACEs[a]**					
0	50252 (61.5)	35190 (64.5)	12208 (57.2)	2161 (46.2)	
1	17667 (21.6)	11375 (20.8)	4846 (22.7)	1146 (24.5)	
2	7343 (9.0)	4470 (8.2)	2180 (10.2)	601 (12.9)	
3	3257 (4.0)	1889 (3.5)	1008 (4.7)	335 (7.2)	
≥4	3228 (3.9)	1668 (3.1)	1086 (5.1)	433 (9.3)	<0.001

Abbreviation: PSU, problematic smartphone use; ACEs, adverse childhood experiences; SD, standard deviation.

[a]: Missing data: 681 for ethnicity, 708 for place of residence, 487 for PSU, 216 for sexual abuse, 219 for physical abuse, 248 for emotional abuse, 325 for physical neglect, and 328 for emotional neglect. 2929 for parental separation or divorce, 2939 for household criminality, 2933 for household domestic violence, 2938 for household mental illness, 2938 for household substance abuse, 2937 for family financial problems, 2938 for death of parents, 2932 for witness of community violence, 2936 for and sex discrimination, 3299 for ACEs score, 1514 for gender expression.

[b] "Others" in ethnicity refers to Miao, Hui, Yi, Dai, and other ethnic groups

© 2024 Zheng X et al. *JAMA Network Open.*

[*]: To compare characteristics across different sexual orientation and gender expression groups, we used analysis of variance for continuous variables and applied Chi-square tests for categorical variables.

© 2024 Zheng X et al. *JAMA Network Open.*

eTable 2. Characteristics of the Included and Excluded Participants

Characteristics	Included, n (%)[a]	Excluded, n (%)[b]
**N**	61761	23285
**Age, mean (SD)**	15.19 (1.71)	14.21 (1.72)
**Sex**		
Male	30941 (50.1)	11473 (49.3)
Female	30820 (49.9)	11812 (50.7)
**Ethnicity**		
Han	51511 (83.4)	18645 (82.3)
Others	10250 (16.6)	3958 (17.7)
**Place of residence**		
Urban	28791 (46.6)	10392 (46.0)
Rural	32970 (53.4)	12186 (54.0)
**ACEs**		
**ACE category: Abuse**		
ACE not reported	56467 (91.4)	21092 (92.1)
ACE reported	5294 (8.6)	1818 (7.9)
**ACE category: Neglect**		
ACE not reported	52380 (84.8)	18461 (80.6)
ACE reported	9381 (15.2)	4431 (19.4)
**ACE category: Household dysfunction**		
ACE not reported	43851 (71.0)	15405 (75.7)
ACE reported	17910 (29.0)	4938 (24.3)

Abbreviation: PSU, problematic smartphone use; ACEs, adverse childhood experiences; SD, standard deviation.

[a]: The included group consisted of participants who had complete data on demographics, PSU, ACEs, sexual orientation, and gender expression variables.

[b]: Missing data of excluded participants: 682 for ethnicity, 707 for place of residence, 375 for abuse, 393 for neglect, and 2942 for household dysfunction.

© 2024 Zheng X et al. *JAMA Network Open.*

eTable 3. Prevalence Estimates of PSU and 95% CIs by Study Characteristics

Characteristics	PSU*		
	Overall	Non-PSU, % (95% CI)	PSU, % (95% CI)
**Total**		64.6 (58.8-69.9)	35.4 (30.0-41.2)
**Age, mean(SD)**	15.12 (0.43)	14.92 (0.45)	15.50 (0.35)
**Sex**			
Male	49.0 (45.0-52.9)	62.6 (56.4-68.4)	37.4 (31.7-43.6)
Female	51.0 (47.1-55.0)	66.5 (61.0-71.7)	33.5 (28.3-39.1)
**Sexual Orientation**			
Heterosexual	83.1 (80.1-85.7)	62.5 (57.4-67.7)	37.5 (30.6-50.3)
Homosexual	2.0 (1.7-2.2)	60.0 (49.7-69.4)	40.1 (30.6-42.3)
Bisexual	9.3 (8.3-10.5)	61.9 (57.7-66.0)	38.1 (34.0-42.3)
Not sure	5.6 (4.0-7.8)	71.2 (64.8-77.0)	28.8 (23.1-35.2)
**Gender Nonconformity**			
Low	70.6 (67.3-73.8)	65.0 (59.2-70.3)	35.1 (29.8-40.8)
Moderate	23.9 (21.6-26.5)	64.1 (58.2-69.6)	35.9 (30.4-41.8)
High	5.4 (4.6-6.4)	60.2 (52.6-67.4)	39.8 (32.6-47.4)
**ACEs**			
**ACE category[a]**			
Abuse reported	8.1 (7.4-8.8)	49.0 (44.0-53.9)	51.0 (46.1-56.0)
Neglect reported	15.0 (12.8-17.4)	57.5 (52.6-62.2)	42.5 (37.8-47.4)
Household dysfunction reported	27.2 (25.0-29.6)	54.8 (50.3-59.3)	45.2 (40.7-49.7)
**Number of ACEs**			
0	4.0 (3.2-5.1)	67.2 (63.4-70.1)	32.8 (29.2-36.6)
1	59.5 (57.9-61.0)	69.8 (64.0-75.3)	30.2 (24.7-36.2)
2	21.6 (20.8-22.4)	58.4 (53.1-63.5)	41.6 (36.5-46.9)

© 2024 Zheng X et al. *JAMA Network Open.*

eTable 3. Prevalence estimates of PSU and 95% confidence intervals (CIs) by study characteristics (continued)

3	8.6 (7.8-9.5)	47.3 (44.0-50.6)	52.7 (49.4-56.0)
≥4	6.3 (5.6-7.1)	64.4 (58.6-69.8)	35.6 (30.3-41.4)

Abbreviation: PSU, problematic smartphone use; ACEs, adverse childhood experiences; SD, standard deviation; 95% CI, 95% confidence interval.

[*]: PSU was measured by the SAS-SV scores, ranging from 10 to 60; male students with SAS-SV scores ≥ 31 and female students with SAS-SV scores ≥ 33 were classified as the PSU group.

[a]: Students who reported any of those abuse (sexual abuse, physical abuse, and emotional abuse) were included in the abuse reported group, and students who reported any of those neglect (physical neglect and emotional neglect) were included in the neglect reported group, students who reported any of those household dysfunction (parental separation or divorce, household criminality, household domestic violence, household mental illness, household substance abuse, family financial problems, death of parent, witness of community violence, and sex discrimination) was included into the household dysfunction reported group.

© 2024 Zheng X et al. *JAMA Network Open.*

eTable 4. Categorized ACEs Across Sexual Orientation and Gender Expressions

ACEs	Sexual Orientation[a]	ACEs	Gender Nonconformity[b]
	AOR (95%CI)*		AOR (95% CI)*
**Each ACE component**		**Each ACE component**	
**Sexual abuse (yes vs. no)**		**Sexual abuse (yes vs. no)**	
Heterosexual	1 (Ref.)	Low	1 (Ref.)
Homosexual	7.27 (4.35-11.55)	Moderate	1.21 (0.98-1.49)
Bisexual	2.97 (2.07-4.20)	High	2.61 (1.92-3.49)
Not sure	0.80 (0.41-1.42)		
**Physical abuse (yes vs. no)**		**Physical abuse (yes vs. no)**	
Heterosexual	1 (Ref.)	Low	1 (Ref.)
Homosexual	4.58 (3.12-6.51)	Moderate	1.65 (1.39-1.96)
Bisexual	2.47 (1.94-3.14)	High	2.81 (2.15-3.62)
Not sure	1.23 (0.86-1.73)		
**Emotional abuse (yes vs. no)**		**Emotional abuse (yes vs. no)**	
Heterosexual	1 (Ref.)	Low	1 (Ref.)
Homosexual	3.08 (2.26-4.12)	Moderate	1.49 (1.31-1.69)
Bisexual	1.90 (1.61-2.25)	High	2.93 (2.41-3.53)
Not sure	0.78 (0.58-1.02)		
**Physical neglect (yes vs. no)**		**Physical neglect (yes vs. no)**	
Heterosexual	1 (Ref.)	Low	1 (Ref.)
Homosexual	2.64 (1.96-3.49)	Moderate	1.51 (1.35-1.68)
Bisexual	1.75 (1.47-2.06)	High	2.09 (1.74-2.50)
Not sure	1.03 (0.82-1.29)		
**Emotional neglect (yes vs. no)**		**Emotional neglect (yes vs. no)**	
Heterosexual	1 (Ref.)	Low	1 (Ref.)

© 2024 Zheng X et al. *JAMA Network Open.*

eTable 4. Categorized ACEs across sexual orientation and gender expressions (continued)

Homosexual	2.37 (1.82-3.04)	Moderate	1.59 (1.45-1.74)
Bisexual	1.43 (1.24-1.66)	High	2.56 (2.20-2.97)
Not sure	1.44 (1.21-1.70)		
**Parental separation or divorce (yes vs. no)**		**Parental separation or divorce (yes vs. no)**	
Heterosexual	1 (Ref.)	Low	1 (Ref.)
Homosexual	1.83 (1.32-2.49)	Moderate	1.18 (1.05-1.33)
Bisexual	1.40 (1.19-1.65)	High	1.51 (1.22-1.84)
Not sure	0.74 (0.57-0.95)		
**Household criminality (yes vs. no)**		**Household criminality (yes vs. no)**	
Heterosexual	1 (Ref.)	Low	1 (Ref.)
Homosexual	0.84 (0.30-1.85)	Moderate	1.16 (0.91-1.48)
Bisexual	1.05 (0.71-1.53)	High	1.90 (1.27-2.74)
Not sure	0.97 (0.60-1.51)		
**Household domestic violence (yes vs. no)**		**Household domestic violence (yes vs. no)**	
Heterosexual	1 (Ref.)	Low	1 (Ref.)
Homosexual	2.35 (1.78-3.06)	Moderate	1.34 (1.21-1.49)
Bisexual	1.60 (1.38-1.85)	High	1.81 (1.50-2.15)
Not sure	0.88 (0.70-1.09)		
**Household mental illness (yes vs. no)**		**Household mental illness (yes vs. no)**	
Heterosexual	1 (Ref.)	Low	1 (Ref.)
Homosexual	2.86 (1.45-5.10)	Moderate	1.55 (1.20-2.01)
Bisexual	2.21 (1.56-3.09)	High	1.45 (0.84-2.33)
Not sure	1.22 (0.68-2.02)		
**Household substance abuse (yes vs. no)**		**Household substance abuse (yes vs. no)**	
Heterosexual	1 (Ref.)	Low	1 (Ref.)

© 2024 Zheng X et al. *JAMA Network Open.*

eTable 4. Categorized ACEs across sexual orientation and gender expressions (continued)

Homosexual	4.73 (2.38-8.51)	Moderate	1.55 (1.12-2.13)
Bisexual	3.01 (1.96-4.51)	High	2.74 (1.66-4.31)
Not sure	1.67 (0.89-2.89)		
**Family financial problems (yes vs. no)**		**Family financial problems (yes vs. no)**	
Heterosexual	1 (Ref.)	Low	1 (Ref.)
Homosexual	1.96 (1.26-2.92)	Moderate	1.30 (1.12-1.51)
Bisexual	1.55 (1.23-1.93)	High	1.90 (1.47-2.43)
Not sure	0.97 (0.70-1.34)		
**Death of parent (yes vs. no)**		**Death of parent (yes vs. no)**	
Heterosexual	1 (Ref.)	Low	1 (Ref.)
Homosexual	1.27 (0.57-2.42)	Moderate	1.15 (0.92-1.43)
Bisexual	1.46 (1.05-1.99)	High	0.78 (0.44-1.27)
Not sure	0.85 (0.50-1.36)		
**Witness of community violence (yes vs. no)**		**Witness of community violence (yes vs. no)**	
Heterosexual	1 (Ref.)	Low	1 (Ref.)
Homosexual	2.07 (1.46-2.86)	Moderate	1.30 (1.15-1.48)
Bisexual	2.09 (1.77-2.47)	High	1.82 (1.47-2.23)
Not sure	0.99 (0.76-1.28)		
**Sex discrimination (yes vs. no)**		**Sex discrimination (yes vs. no)**	
Heterosexual	1 (Ref.)	Low	1 (Ref.)
Homosexual	4.14 (3.03-5.56)	Moderate	1.52 (1.32-1.75)
Bisexual	2.37 (1.99-2.81)	High	2.98 (2.40-3.67)
Not sure	1.12 (0.84-1.48)		

Abbreviation: ACEs, adverse childhood experiences; AOR, adjusted odds ratio; 95% CI, 95% confidence interval; Ref., reference.

*: Models were adjusted for age, sex, ethnicity, and place of residence.

© 2024 Zheng X et al. *JAMA Network Open.*

[a]: Heterosexual=male students who describe themselves as romantically attracted to females; female students who describe themselves as romantically attracted to males. Homosexual=male students who describe themselves as romantically attracted to males; female students who describe themselves as romantically attracted to females. Bisexual=students who describe themselves as romantically attracted to both males and females. In this analysis, the variable of sexual orientation was categorized into heterosexual, homosexual, bisexual, and not sure adolescents, and the reference group was heterosexual adolescents.

[b]: Low=Female students who describe themselves as very/mostly/somewhat feminine; male students who describe themselves as very/mostly/somewhat masculine. Moderate=Students who describe themselves as equally feminine and masculine. High=Female students who describe themselves as very/mostly/somewhat masculine; male students who describe themselves as very/mostly/somewhat feminine. In this analysis, the variable of gender nonconformity was categorized into three groups: low GNC, moderate GNC, and high GNC adolescents. The reference group was low GNC adolescents.

© 2024 Zheng X et al. *JAMA Network Open.*

eTable 5. Interactions Between ACE and Sexual Orientation/Gender Expression on PSU[a]

	ACE×Sexual orientation				ACE×Gender expression			
	**Model 1**		**Model 2**		**Model 1**		**Model 2**	
	**PR (95% CI)**	***P*-value**	**APR (95% CI)**	***P*-value**	**PR (95% CI)**	***P*-value**	**APR (95% CI)**	***P*-value**
**PSU[b]**	1.00 (0.99-1.02)	0.51	1.00 (0.99-1.02)	0.78	0.98 (0.97-0.99)	0.03	0.98 (0.97-0.99)	0.03

Abbreviation: PSU, problematic smartphone use; ACEs, adverse childhood experiences; PR, prevalence ratio; APR, adjusted prevalence ratio; 95% CI, 95% confidence interval.

[a]: Models showed good model fit, with the Wald test reporting $P<0.001$ and the Pearson goodness-of-fit test reporting $P>0.10$.

[b]: PSU was measured by the SAS-SV scores, ranging from 10 to 60; male students with SAS-SV scores ≥ 31 and female students with SAS-SV scores ≥ 33 were classified as the PSU group.

Model 1 was a crude model.

Model 2 was adjusted for age, sex, ethnicity, and place of residence.

© 2024 Zheng X et al. *JAMA Network Open.*

eReferences

1. Lai W, Li W, Guo L, et al. Association between bullying victimization, coping style, and mental health problems among Chinese adolescents. *J Affect Disorders*. 2023;324:379-386. doi:10.1016/j.jad.2022.12.080

2. Guo L, Wang W, Gao X, Huang G, Li P, Lu C. Associations of Childhood Maltreatment with Single and Multiple Suicide Attempts among Older Chinese Adolescents. *J Pediatr-Us*. 2018;196:244-250.e1. doi:10.1016/j.jpeds.2018.01.032

3. Bernstein DP, Stein JA, Newcomb MD, et al. Development and validation of a brief screening version of the Childhood Trauma Questionnaire. *Child Abuse Neglect*. 2003;27(2):169-90. doi:10.1016/s0145-2134(02)00541-0

4. Xie P, Wu K, Zheng Y, et al. Prevalence of childhood trauma and correlations between childhood trauma, suicidal ideation, and social support in patients with depression, bipolar disorder, and schizophrenia in southern China. *J Affect Disorders*. 2018;228:41-48. doi:10.1016/j.jad.2017.11.011

5. Peng C, Cheng J, Rong F, Wang Y, Yu Y. Psychometric properties and normative data of the childhood trauma questionnaire-short form in Chinese adolescents. *Front Psychol*. 2023;14:1130683. doi:10.3389/fpsyg.2023.1130683

6. Huang C, Yuan Q, Ge M, et al. Childhood Trauma and Non-suicidal Self-Injury Among Chinese Adolescents: The Mediating Role of Psychological Sub-health. *Front Psychiatry*. 2022;13:798369. doi:10.3389/fpsyt.2022.798369

7. Felitti VJ, Anda RF, Nordenberg D, et al. Relationship of childhood abuse and household dysfunction to many of the leading causes of death in adults. The Adverse Childhood Experiences (ACE) Study. *Am J Prev Med*. 1998;14(4):245-58. doi:10.1016/s0749-3797(98)00017-8

8. Zhao M, Xiao D, Wang W, et al. Association of sexual minority status, gender nonconformity with childhood victimization and adulthood depressive symptoms: A path analysis. *Child Abuse Neglect*. 2021;111:104822. doi:10.1016/j.chiabu.2020.104822

9. Wylie SA, Corliss HL, Boulanger V, Prokop LA, Austin SB. Socially assigned gender nonconformity: A brief measure for use in surveillance and investigation of health disparities. *Sex Roles*. 2010;63(3-4):264-276. doi:10.1007/s11199-010-9798-y

10. Gordon AR, Austin SB, Schultz J, Guss CE, Calzo JP, Wang ML. Gender Expression, Peer Victimization, and Disordered Weight-Control Behaviors Among U.S. High School Students. *J Adolescent Health*. 2021;68(6):1148-1154. doi:10.1016/j.jadohealth.2020.08.032

11. Lowry R, Johns MM, Gordon AR, Austin SB, Robin LE, Kann LK. Nonconforming Gender Expression and Associated Mental Distress and Substance Use Among High School Students. *Jama Pediatr*. 2018;172(11):1020-1028. doi:10.1001/jamapediatrics.2018.2140

12. Zheng X, Yang Y, Jiang W, et al. Nonconforming gender expression and associated problematic smartphone and internet use among Chinese adolescents. *J Behav Addict*. 2023;12(3):817-26. doi:10.1556/2006.2023.00040

© 2024 Zheng X et al. *JAMA Network Open.*
