# Supplementary material for: Childhood Adversity and Adolescent Smartphone Use Across Sexual Orientation and Gender Expression
Source: JAMA Netw Open. 2024 Apr 12;7(4):e246448. doi: 10.1001/jamanetworkopen.2024.6448 (PMC11015349; doi:10.1001/jamanetworkopen.2024.6448)
Supplement: Supplement 2. — Data Sharing Statement [file jamanetwopen-e246448-s002.pdf]

Data Sharing Statement

Zheng. Childhood Adversity and Adolescent Smartphone Use Across Sexual Orientation and Gender Expression. *JAMA Netw Open*. Published April 12, 2024.
doi:10.1001/jamanetworkopen.2024.6448

Data
Data available: No

Additional Information
Explanation for why data not available: Data are not publicly available due to their containing information that could compromise research participant privacy/consent. Data will be made available only to potential collaborators with ethical approval after they submit a research proposal application by contacting the corresponding authors.
